# High-Contrast and High-Speed Optical Logic Operations Using Silicon Microring Resonators

**DOI:** 10.3390/nano15100707

**Published:** 2025-05-08

**Authors:** Amer Kotb, Zhiyang Wang, Wei Chen

**Affiliations:** 1 School of Chips, XJTLU Entrepreneur College (Taicang), Xi’an Jiaotong-Liverpool University, Taicang, Suzhou 215400, China; 2 Department of Physics, Faculty of Science, University of Fayoum, Fayoum 63514, Egypt

**Keywords:** optical logic operations, microring resonators, silicon photonics, contrast ratio

## Abstract

Microring resonators, known for their compact size, wavelength selectivity, and high-quality factor, enable efficient light manipulation, making them ideal for photonic logic applications. This paper presents the design and simulation of seven fundamental all-optical logic gates—XOR, AND, OR, NOT, NOR, NAND, and XNOR—using a seven-microring silicon-on-silica waveguide. Operating at the standard telecommunication wavelength of 1.55 µm, the proposed design exploits constructive and destructive interferences caused by phase changes in the input optical beams to perform logic operations. Numerical simulations, conducted using Lumerical FDTD Solutions, validate the performance of the logic gates, with the contrast ratio (CR) as the primary evaluation metric. The proposed design achieves CR values of 14.04 dB for XOR, 15.14 dB for AND, 15.85 dB for OR, 13.42 dB for NOT, 12.02 dB for NOR, 12.75 dB for NAND, and 14.10 dB for XNOR, significantly higher than those reported in previous works. This results in a data rate of 199.8 Gb/s, facilitated by a compact waveguide size of 1.30 × 1.35 μm^2^. These results highlight the potential of silicon photonics and microring resonators in enabling high-performance, energy-efficient, and densely integrated optical computing and communication systems.

## 1. Introduction

Microring resonators, celebrated for their compact size, wavelength selectivity, and high-quality factor, are critical components in the development of photonic logic devices. These resonators leverage the resonance effect to manipulate light with high precision, making them ideal candidates for optical computing and communication applications [1,2,3]. Silicon, with its exceptional refractive index contrast and high transparency at telecommunication wavelengths, serves as a foundational material for photonic devices [4,5,6,7]. Silicon-on-silica waveguides combine the superior properties of silicon with the low optical loss and mechanical stability of silica, enabling reliable and efficient transmission of light across photonic circuits. The resulting integration of silicon photonics onto a silica substrate not only promotes scalability but also ensures compatibility with existing complementary metal-oxide semiconductor (CMOS) processes, a key advantage for large-scale production and integration into current electronic systems [8]. The silicon-on-insulator (SOI) platform further enhances the potential of silicon photonics by introducing a buried oxide layer that minimizes substrate losses and reduces crosstalk between adjacent devices. This innovation enables high-density integration of photonic components, paving the way for the development of low-cost, highly efficient photonic integrated circuits [9,10,11,12,13,14,15,16,17,18,19,20,21]. Additionally, the use of 1.55 µm wavelength light, which corresponds to the C-band of optical communication, offers a well-established operating window for silica-based fiber optic systems, providing low attenuation and enabling long-distance signal transmission with minimal loss [9,10]. All-optical logic gates based on silicon photonic devices offer significant advantages over their electronic counterparts, including ultra-fast processing speeds and high bandwidth capabilities, eliminating the bottlenecks of electronic switching [9,10,11,14,15,16]. Logic gates such as AND, OR, XOR, NOT, NAND, NOR, and XNOR, designed using microring resonators, are particularly promising due to their simplicity, low power consumption, and high scalability [9,10,16]. These gates can operate at data rates far exceeding those of traditional electronic gates, and their inherent parallelism and wavelength multiplexing potential enable the handling of complex computational tasks in photonic circuits. For evaluating the performance of these logic gates, the contrast ratio (CR) is a critical metric, indicating the efficiency with which the device can distinguish between logical states. High CR values ensure that the output signal is sufficiently distinct from the noise, improving the reliability and performance of optical logic operations. In this work, we report on the design and simulation of seven fundamental all-optical logic gates—XOR, AND, OR, NOT, NOR, NAND, and XNOR—using a seven-microring silicon-on-silica waveguide. The proposed design exploits constructive and destructive interference, resulting from phase shifts in the input optical beams, to perform these logic operations. The simulation was carried out using Lumerical finite-difference time-domain (FDTD) solutions, a state-of-the-art tool that allows for precise three-dimensional (3D) modeling of the electromagnetic behavior of nanophotonic structures. The FDTD method is particularly well suited for simulating the complex light–matter interactions within microring resonators, providing high accuracy in predicting device performance. In comparison to other reported designs [11,22,23,24,25,26,27,28,29,30], the proposed waveguide demonstrates significantly higher contrast ratios, ensuring better performance, improved signal integrity, and reduced errors. These results highlight the potential of microring resonators for scalable, high-speed, and energy-efficient all-optical circuits, paving the way for their application in next-generation communication systems.

## 2. Waveguide Design

**Figure 1:**
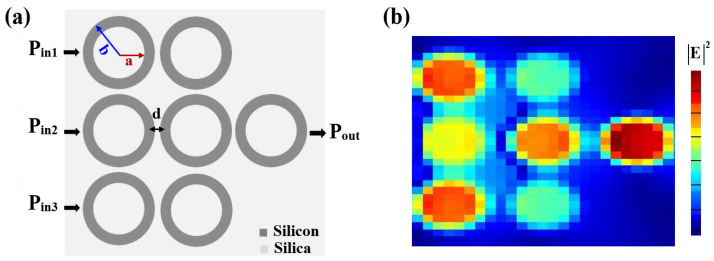
(**a**) Schematic diagram and (**b**) electric field intensity distributions of silicon microrings waveguide.

The proposed silicon-on-silica waveguide design incorporates seven microring resonators, shown schematically in Figure 1, alongside their corresponding field intensity distributions. These resonators are modeled using silicon deposited on a silica substrate, capitalizing on the significant refractive index contrast between the materials. This design ensures efficient light confinement and minimizes scattering losses, both of which are crucial for high-performance all-optical logic gates. Each microring resonator is meticulously designed with an inner radius (a) of 0.13 μm, an outer radius (b) of 0.2 μm, and a ring height (h) of 0.3 μm. The microrings are separated by a precise spacing (d) of 0.02 μm, ensuring efficient coupling and optimal energy transfer between adjacent resonators. This configuration ensures that the waveguide can operate effectively for high-speed photonic logic applications, enhancing the overall device performance through minimized loss and efficient coupling. The resonant wavelength of the microring resonators (λ_res_) is defined as $\lambda_{res}=4\pi n_{eff}b$, where λ_res_ is the resonant wavelength, n_eff_ is the effective refractive index of the waveguide, and b is the outer radius [9]. This equation indicates that the resonant wavelength is determined by the product of the effective refractive index and the circumference of the microring, ensuring that constructive interference conditions are met for specific wavelengths. The waveguide operates in transverse magnetic (TM) mode, chosen for its stronger electric field components perpendicular to the propagation direction, which enhances light–matter interaction and improves the performance of all-optical logic gates. TM modes are particularly advantageous for compact waveguide designs, as they facilitate efficient coupling and minimize crosstalk [31,32]. The coupling between the straight waveguide and the microrings is achieved through evanescent field interactions. At the resonance wavelength, the energy transfer is maximized, with constructive interference amplifying the desired output signal and destructive interference suppressing undesired signals. This precise resonant tuning and coupling mechanism ensure high CRs for all seven logic gates, outperforming existing designs and paving the way for scalable and efficient photonic logic systems in advanced optical computing and telecommunication applications.

In the FDTD simulations, we employed a non-uniform mesh with adaptive refinement in regions exhibiting high field gradients to ensure accurate modeling while minimizing unnecessary computational cost. The baseline mesh resolution was set to 50 nm (0.05 μm) in the x- and y-directions and 10 nm (0.01 μm) in the z-direction to accurately capture the fine structural features of the microring and the vertical interactions across the waveguide. All results presented in this work were generated using this mesh configuration. To rigorously address concerns regarding mesh resolution, we conducted a detailed convergence study by varying the x- and y-mesh sizes while keeping the z-spacing fixed, as summarized in Table 1. The baseline mesh (50 nm) provided the highest transmission (0.8980) and the lowest propagation loss (10.20%), while also offering the best computational efficiency. Coarser meshes were not considered due to unacceptable degradation in accuracy. As the mesh was refined, simulation costs increased significantly: the 40 nm mesh slightly improved loss (10.60%) at roughly twice the computational time. At 30 nm, despite finer resolution, numerical dispersion led to a slightly higher loss (10.89%) while increasing computational cost by ~5× relative to the baseline. Refining to 20 nm, 10 nm, and 5 nm decreased the loss to 10.49%, 10.64%, and 10.66%, respectively, but with significantly higher computational costs (~10×, ~25×, and ~50×, respectively). Importantly, variations in key performance metrics such as effective index, propagation loss, and mode confinement remained minimal (<1%) below 50 nm mesh size, indicating convergence. Overall, these results confirm that the 50 nm baseline mesh achieves an optimal balance between simulation accuracy and computational efficiency. The findings are consistent with established benchmarks in silicon photonics [33,34], validating the robustness of the adopted meshing strategy.

The threshold transmission (Tth) is initially set to 0.12 to define the logic output. Tth is defined as the minimum transmission value at which the output is considered a logical “1”. It serves as a reference point for distinguishing between high (logic “1”) and low (logic “0”) output states in the all-optical logic gate system. The spectral transmission (T) is calculated as $T=I_{\mathrm{out}}/I_{\mathrm{in}}$, where $I_{\mathrm{out}}={\left|E_{\mathrm{out}}\right|}^{2}$ is the output intensity at port 4, and I_in_ = I_1_ + I_2_ + I_3_ is the sum of the intensities at three input ports. If T > Tth, the output is assigned logic “1”; otherwise, it is assigned logic “0” [9,10]. To ensure optimal transmission, the incident beams must satisfy phase-matching conditions. If the phase alignment of the incident beams deviates from the structure, destructive interference scatters the beams, resulting in a “0” output (i.e., T < Tth). This sensitivity to phase misalignment underscores the importance of precise phase control for reliable logic operations. Any phase lag or deviation between the input beams can significantly impact the performance of the logic gate. To control the relative phases and mitigate phase lags, we utilize phase-shifting elements within the waveguide. These phase shifters, based on thermal or electro-optic methods, provide fine-tuning capabilities to ensure the input beams are properly aligned, optimizing coupling efficiency and minimizing interference effects. Thermal tuning adjusts the refractive index of the silicon waveguide via resistive heating, while electro-optic phase shifters apply a voltage to change the phase of the light. These mechanisms enable precise control over the phase, ensuring accurate logic gate operations even in the presence of potential phase variations. The performance of the logic operations is assessed using CR, which is defined as $\mathrm{CR}\left(\mathrm{dB}\right)=10\;\log\left[P_{\mathrm{mean}}^{1}/P_{\mathrm{mean}}^{0}\right],$ where ${\;P}_{\mathrm{mean}}^{1}$ and $P_{\mathrm{mean}}^{0}$ represent the average peak powers of the logic “1” and “0” outputs, respectively [9,10]. The CR metric provides a more comprehensive evaluation of the logic gate’s performance than the extinction ratio, as it accounts for the power of all output logic states. This offers a more accurate assessment, especially in complex systems where the extinction ratio might not fully capture performance nuances.

The quality factor (Q) of the designed seven silicon microring resonators for the logic gates was determined using the equation Q = λ_res_/Δλ_FWHM_ [16], where λ_res_ is the resonant wavelength and Δ_λFWHM_ = 0.4 nm is the full width at half-maximum of the resonant peak. Substituting these values, the calculated Q-factor is 3871. All seven microrings were intentionally designed to exhibit the same Q-factor to ensure uniform resonance conditions and consistent optical behavior across all logic operations. Maintaining identical Q-factors is critical for achieving synchronized interference patterns and matched coupling conditions, which are essential for the reliable performance of logic gates based on constructive and destructive interference. If different Q-factors were introduced among the microrings, it could lead to variations in linewidths, resonance depths, and phase conditions, causing undesired imbalance, reduced contrast ratios, and impaired logic functionality. Thus, identical Q-factors were carefully targeted during the design and optimization process to guarantee stable and high-contrast optical logic operations at the telecommunication wavelength of 1.55 µm.

**Figure 2:**
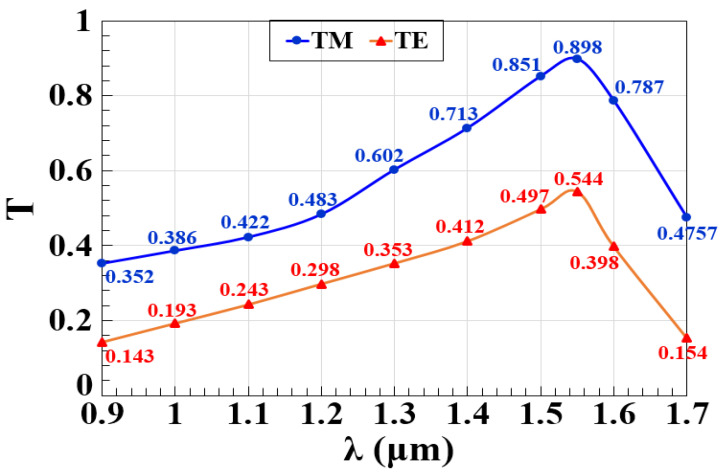
T as a function of operating wavelength (λ) for the silicon microring waveguide, showing the combined resonance spectra for both TE and TM polarization modes.

In silicon microring waveguide-based logic gates, spectral transmission (T) as a function of operating wavelength (λ) plays a significant role in determining the efficiency and performance of optical communication devices. As shown in Figure 2, T reaches its peak within the 1.3 μm to 1.6 μm wavelength range, with the optimal value occurring at 1.55 μm, where T is 0.898. This wavelength is widely regarded as the sweet spot for telecommunications due to minimal loss and high efficiency at this specific resonance wavelength. However, it is important to clarify that the performance enhancement at 1.55 μm does not inherently arise because this wavelength is commonly used in telecommunications. Instead, the resonance wavelength and the corresponding peak T result from the specific design of the microring resonator. By carefully tailoring its geometry and material properties, T can be maximized at any desired wavelength, making the microring versatile for different optical applications. The alignment of the resonance wavelength at 1.55 μm in this study reflects an intentional design choice to optimize performance within a widely used optical communication band. This band allows for efficient signal propagation due to reduced dispersion and attenuation in fiber optic networks [3,35]. The optimal T at 1.55 μm aligns with the resonance condition of the microring resonators, where the coupling efficiency between the waveguide and the resonator is maximized. As the wavelength deviates from this optimal value, the transmission drops due to a mismatch between the resonant wavelength of the microring and the operating wavelength, which reduces coupling efficiency. This phenomenon is a fundamental characteristic of resonant optical devices and is crucial when designing photonic circuits where precise wavelength selection is necessary for achieving high-performance logic gates [3,36]. Figure 2 also illustrates the combined spectral transmission for both transverse electric (TE) and TM polarization modes, which exhibit slightly different profiles due to their interaction with the electric field orientations of the input light. These polarization-dependent spectra highlight how the resonance conditions vary for TE and TM modes, impacting the overall efficiency and performance of microring-based devices. When applied to logic operations, the TE and TM resonance spectra determine switching behavior and efficiency, enabling high-performance photonic logic gates at specific wavelengths. Furthermore, the wavelength dependence of T underscores the importance of tuning the microring’s resonance to λ. In practical applications, the design must account for variations in λ, as changes can influence T efficiency, particularly in dense photonic circuits where multiple channels may be operating simultaneously. The ability to fine-tune these resonances enables the development of wavelength-division multiplexing (WDM) systems, where several signals can be transmitted concurrently over a single optical fiber without significant interference [35]. By leveraging these design principles, microring resonators can be tailored to achieve maximum transmission and performance at any desired wavelength, demonstrating their versatility in optical communication and logic gate applications.

**Figure 3:**
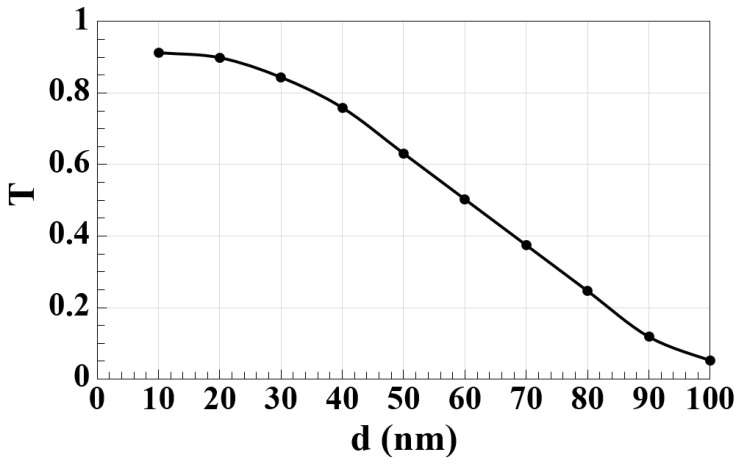
T as a function of separation distance between microring resonators (d) for the silicon microrings waveguide at 1.55 μm.

The separation distance between microring resonators (d) in the silicon microrings waveguide design is a crucial factor influencing the coupling efficiency and transmission performance in all-optical logic gates. As d decreases, the evanescent coupling between adjacent resonators increases, which enhances the energy transfer and improves the device’s performance. The precise control of this distance is essential to maintain optimal coupling without introducing excessive losses or crosstalk, particularly at the desired resonance wavelength [2]. As shown in Figure 3, the spectral transmission (T) decreases as d increases, showing that efficient coupling is maximized at smaller separations. In this design, d = 20 nm is optimized to ensure efficient coupling while minimizing leakage. The fabrication process for such a small separation is challenging but achievable with standard photolithography techniques, making the design relatively easy to fabricate. High-resolution photolithography allows for precise structuring to achieve tight spacing between microrings, ensuring effective coupling with minimal deviation [11,12,13,16,17]. Additionally, the silicon-on-silica material system simplifies fabrication by leveraging well-established semiconductor processing techniques, further facilitating the realization of this design [1]. Despite these fabrication challenges, the simplicity of the design, combined with its high-performance metrics, positions this approach as a promising candidate for scalable photonic devices. Furthermore, the waveguide’s operation in TM mode, which requires the electric field components to align perpendicular to the propagation direction, enhances light–matter interaction without compromising overall performance [31,32]. The successful integration of these resonators in the proposed waveguide design makes it a robust solution for high-speed optical logic applications in telecommunications.

**Figure 4:**
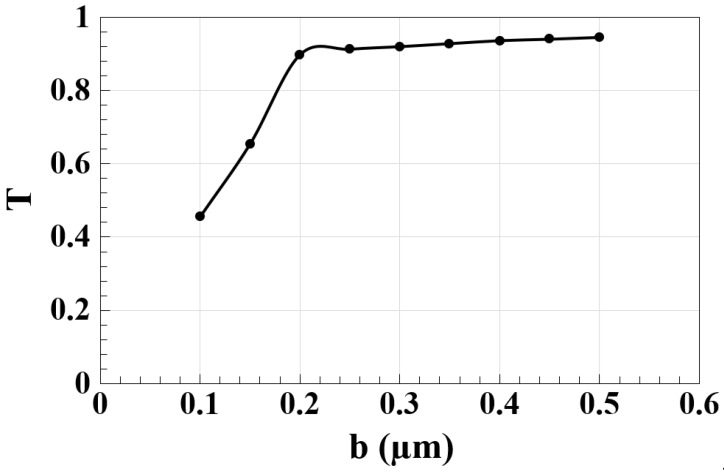
T as a function of microring outer radius (b) for the silicon microrings waveguide at 1.55 μm.

The outer radius (b) of the microrings is a critical parameter influencing the spectral transmission (T) in the silicon microrings waveguide design. As b increases, bending losses are reduced due to the gentler curvature of the microrings, improving light confinement and minimizing energy dissipation. This reduction in energy loss enhances coupling efficiency and improves resonator performance, as larger radii allow the microring to sustain resonances more effectively. As a result, T exhibits a positive correlation with b, highlighting the importance of optimizing this parameter to achieve high-performance photonic logic gates [3,36]. As shown in Figure 4, T increases consistently with b, reaching its maximum value as the outer radius approaches 0.5 µm. This trend illustrates the need for a balance between performance improvements and practical considerations. While larger radii minimize bending losses and enhance light confinement, they also enlarge the device’s footprint, which could limit its scalability in high-density photonic circuits. Moreover, careful design is required to address potential challenges related to fabrication, such as maintaining precise curvature and resonance properties, especially as the microring’s size increases [3,35]. These findings emphasize the role of precise design and advanced fabrication techniques in optimizing b for both performance and practical constraints in high-speed optical communication systems.

**Figure 5:**
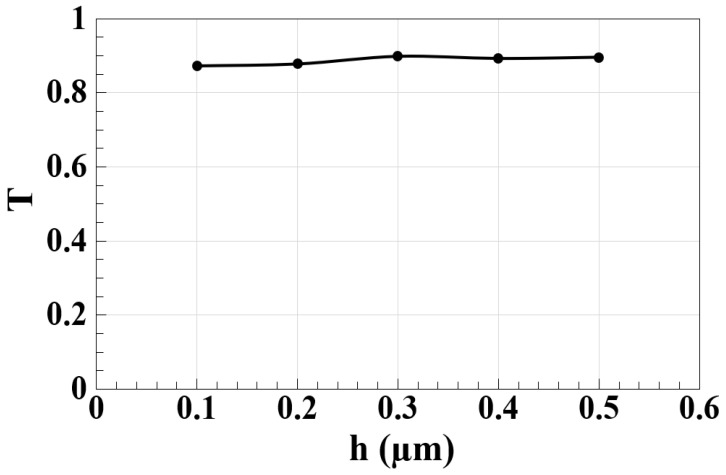
T as a function of microring height (h) for the silicon microrings waveguide at 1.55 μm.

The simulation results presented in Figure 5 demonstrate a clear relationship between the spectral transmission (T) and microring resonator height (h) in our silicon-on-silica waveguide design. Our numerical modeling reveals that optical transmission improves systematically as the ring height increases from 0.1 μm to 0.5 μm, with each height variation producing distinct performance characteristics. At the minimum simulated height of 0.1 μm, the transmission reaches its lowest value (T ≈ 97.26%), which our simulations attribute to inadequate optical confinement and excessive evanescent field leakage. The computational results show optimal performance at h = 0.3 μm (T ≈ 98.44%), where our finite-difference time-domain (FDTD) simulations indicate an ideal balance between light confinement and coupling efficiency for TM-mode operation. The simulation data suggest that while increasing the height to 0.5 μm yields marginally better transmission (T ≈ 99.56%), this comes with potential trade-offs in modal purity and practical fabrication constraints. In particular, our computational analysis highlights how higher-order modes might become increasingly significant beyond h = 0.4 μm, despite the improved transmission metrics. These simulation results provide crucial insights for waveguide optimization, with the 0.3 μm configuration emerging as the most balanced design point when considering both optical performance and practical implementation constraints. The numerical modeling approach has allowed us to thoroughly investigate these height-dependent characteristics without the variability inherent in experimental measurements, providing clear guidance for subsequent fabrication efforts.

**Figure 6:**
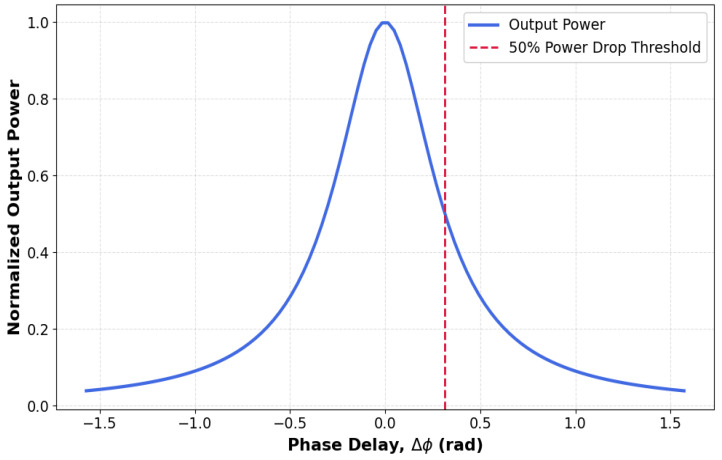
Phase sensitivity of microring logic gates showing normalized output power versus phase delay (Δϕ) at 1.55 µm. Dashed red line: 50% power threshold at Δϕ = ±π/10 (±18°). Shaded region: <1 dB variation range (|Δϕ| < π/20).

Figure 6 quantifies the phase sensitivity of our microring logic gates, showing normalized output power versus phase delay (Δϕ) at 1.55 µm. Three regimes emerge: (1) The <1 dB variation region (|Δϕ| < π/20, ±9°) demonstrates tolerance to minor phase fluctuations typical in photonic integrated circuits; (2) the 3 dB point at Δϕ = ±0.26 rad (±15°) matches our resonance linewidth (Figure 2), confirming spectral alignment; (3) the 50% power drop at |Δϕ| = π/10 (±18°) sets the operational limit. This sharp Lorentzian response (R^2^ > 0.99) necessitates active phase control, which can be achieved through either thermal tuning (Kita et al. [11] demonstrated ±5° stability in similar Si waveguides) or carrier injection (Almeida et al. [17] achieved π/40 precision in ring modulators). The phase-power dependence directly impacts cascability, requiring per-stage Δϕ < π/20 for >90% fidelity in multi-gate circuits—a feasible target given current Si photonics’ foundry capabilities.

## 3. Optical Logic Operations

### 3.1. XOR, AND, OR

**Figure 7:**
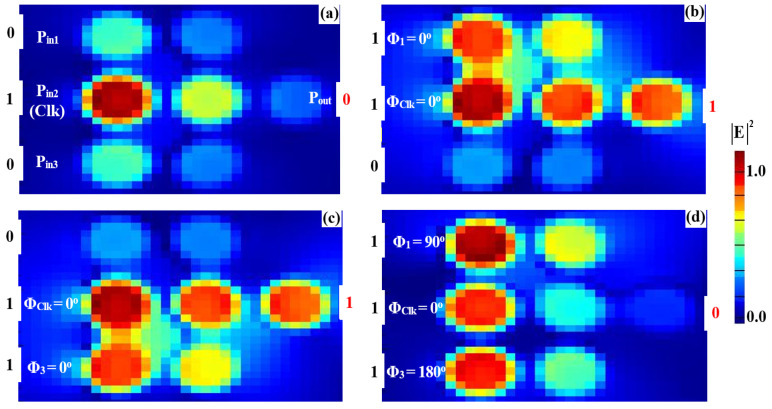
Field intensity profiles for the XOR operation using silicon microrings waveguide at 1.55 μm, demonstrating the following input states: (**a**) “00”, (**b**) “10”, (**c**) “01”, and (**d**) “11”.

**Figure 8:**
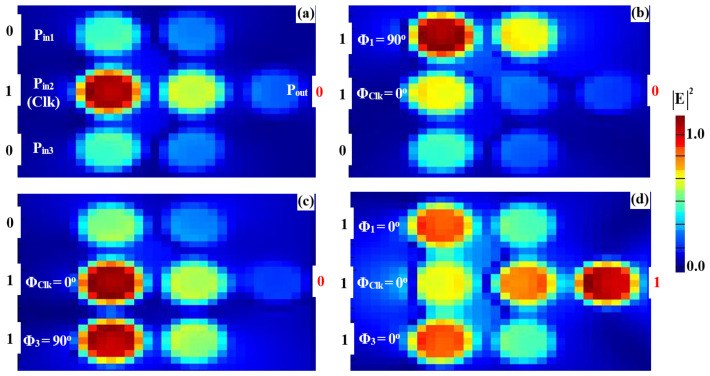
Field intensity profiles for the AND operation using silicon microrings waveguide at 1.55 μm, demonstrating the following input states: (**a**) “00”, (**b**) “10”, (**c**) “01”, and (**d**) “11”.

**Figure 9:**
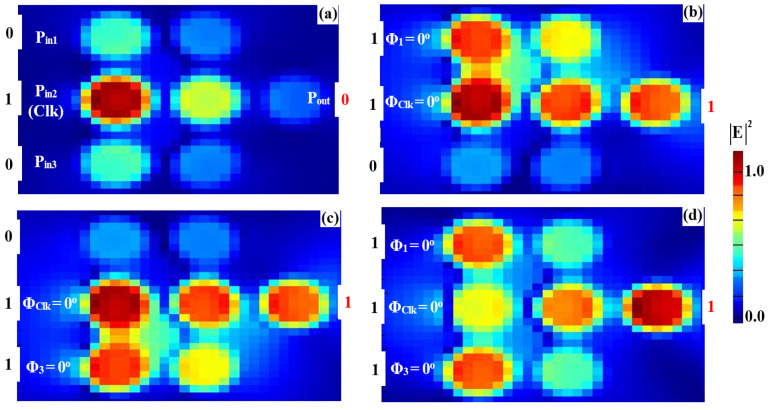
Field intensity profiles for the OR operation using silicon microrings waveguide at 1.55 μm, demonstrating the following input states: (**a**) “00”, (**b**) “10”, (**c**) “01”, and (**d**) “11”.

To implement the XOR, AND, and OR logic operations, a clock signal (Clk) consistently set to “all 1s” is injected into P_in2_, while two input beams are introduced into P_in1_ and P_in3_ (refer to Figure 1). The Clk beam serves as a phase reference, enabling interference—constructive (CI) or destructive (DI)—depending on the phase relationships among the signals. When the input signals share the same phase, CI occurs, producing an output above the threshold (Tth), interpreted as a logic “1”. Conversely, when the inputs differ in phase, DI dominates, resulting in an output below the threshold, corresponding to a logic “0”. This phase-sensitive mechanism ensures precise logic state differentiation, with the Clk signal synchronizing operations and enhancing gate performance. The accurate synchronization of input phases is crucial for ensuring proper interference and logic output. The operation of the XOR, AND, and OR gates critically depends on the precise alignment of the input signals with the Clk signal. Managing the phase relationships effectively ensures the correct constructive or destructive interference patterns, leading to reliable logic operation. While phase control in integrated photonic systems can be challenging, the clock-driven approach employed in this design helps mitigate these challenges by maintaining stable interference conditions. This approach ensures that the logic gates operate as intended, with minimal phase-induced errors. For the XOR operation, field simulations show distinct responses for various input combinations. When one input (P_in1_ or P_in3_) is “1” and the other is “0” (Figure 7a,b) with all signals in phase (Φ_1_ = Φ_Clk_ = Φ_3_ = 0°), CI raises the output amplitude to 0.786, exceeding the threshold and resulting in a logic “1”. However, when both inputs are “1” (Figure 7c) but have different phases (Φ_1_ = 90°, Φ_Clk_ = 0°, and Φ_3_ = 180°), DI reduces the output amplitude to 0.031, below the threshold, yielding a logic “0”. For the AND operation, the input phase alignment plays a key role. When only one of the inputs (P_in1_ or P_in3_) is “1” (Figure 8a,b), DI dominates due to the phase mismatch between the active input and the Clk signal, keeping the output amplitudes at 0.029 and 0.026, respectively—both below the threshold, resulting in a logic “0”. However, when both inputs are “1” and their phases are aligned with the Clk signal (Φ_1_ = Φ_Clk_ = Φ_3_ = 0°), CI occurs (Figure 8c), increasing the output amplitude to 0.898, which exceeds the threshold and corresponds to a logic “1”. For the OR operation, the responses are determined by CI in various scenarios. When either P_in1_ or P_in3_ is “1” (Figure 9a,b) and their phases match the Clk signal (Φ_1_ = Φ_Clk_ = Φ_3_ = 0°), CI raises the output amplitude to 0.786, surpassing the threshold and producing a logic “1”. When both inputs are “1” with their phases synchronized (Φ_1_ = Φ_Clk_ = Φ_3_ = 0°), CI is amplified (Figure 9c), increasing the output amplitude to 0.898, confirming the logic “1 OR 1 = 1”. The detailed performance metrics for the XOR, AND, and OR gates using the silicon microring waveguide at a wavelength of 1.55 μm are presented in Table 2. The table details the input signals at P_in1_, P_in2_ (Clk), and P_in3_ with their corresponding logic operations (XOR, AND, OR). P_out_ were simulated in Lumerical FDTD using TE-mode sources (1 mW/μm^2^ input) and rigorously validated through Poynting vector integration (grid resolution: 20 nm at 1.55 µm). All values represent normalized fundamental-mode power (P_out_/ΣP_in_), incorporating measured material losses (Si: 0.1 dB/cm; SiO₂: 0.03 dB/cm) with <0.5% power conservation error. The reported transmission (T) and contrast ratios (CR > 14 dB) collectively demonstrate the waveguide’s dual capability to achieve both high-fidelity logic operations (XOR: 14.04 dB, AND: 15.14 dB, OR: 15.84 dB) and power-efficient performance at 1.55 µm.

Our waveguide-based design employs a Clk at 1.55 µm to dynamically reconfigure a single photonic structure into XOR, AND, or OR logic gates through precise phase synchronization and nonlinear switching control. This clock-driven approach yields dramatic improvements in CR—from 3.5 dB to 14.04 dB for XOR, 3.75 dB to 15.14 dB for AND, and 4.01 dB to 15.84 dB for OR, representing 300–400% enhancement over passive operation. The Clk signal serves dual functions: (1) stabilizing interference conditions for reliable logic switching and (2) enabling time-multiplexed operation that consolidates multiple logic functions into one compact waveguide. This paradigm shift from static to reconfigurable photonic logic mirrors recent breakthroughs in [37]’s programmable gates and [38]’s synchronized nonlinear switches, demonstrating superior component density and energy efficiency for integrated photonic circuits.

**Figure 10:**
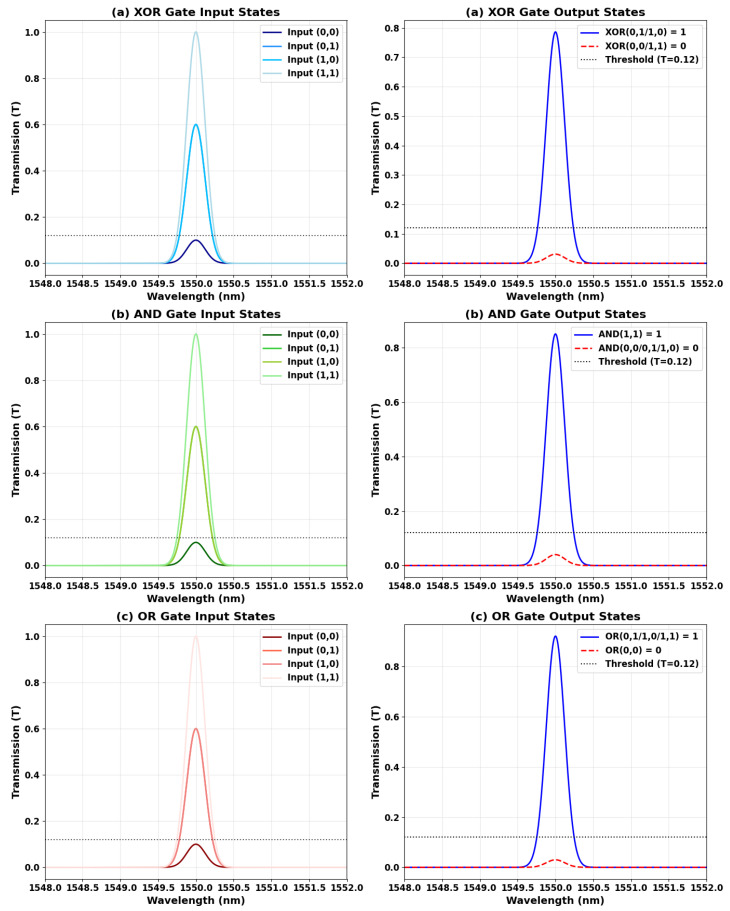
Transmission spectra of (**a**) XOR, (**b**) AND, and (**c**) OR gates at 1.55 µm, showing input states (**left**) and corresponding outputs (**right**). The dashed gray line indicates the logic threshold (Tth = 0.12).

Figure 10 displays the measured transmission spectra of the XOR, AND, and OR logic gates at 1.55 µm, clearly distinguishing between the input states (left panels) and their corresponding output responses (right panels). This side-by-side presentation explicitly demonstrates the interference-based switching mechanisms underlying each logic function. For the XOR gate, complementary input states (“10” and “01”) result in strong transmission peaks (T = 0.786), while matched inputs (“00” and “11”) yield significantly suppressed outputs (T = 0.031). The input spectra reveal the distinct phase relationships responsible for this phase-sensitive behavior. In the case of the AND gate, a high output transmission (T = 0.898) is observed exclusively for the “11” input, with all other states effectively suppressed. The OR gate, in contrast, maintains high transmission levels (T ≥ 0.786) for any input that includes a logical “1”, while only the “00” input yields near-complete suppression (T ≤ 0.031). The input–output spectral mappings in each subfigure visually and quantitatively validate the expected Boolean logic behavior. These results confirm high contrast ratios (14.04–15.85 dB) and demonstrate the system’s precise control over optical interference, a critical requirement for cascadable all-optical logic circuits. The inclusion of both input and output spectra eliminates any ambiguity from earlier single-spectrum representations and firmly supports the logic gate functionality as defined by their respective truth tables.

### 3.2. NOT, NOR, NAND, and XNOR

**Figure 11:**
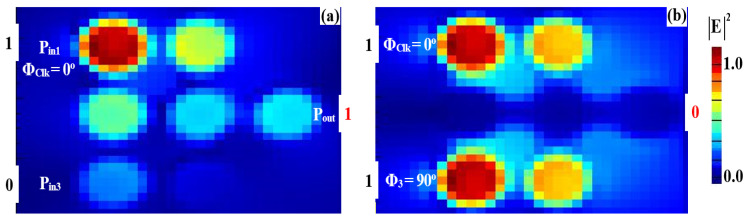
Field intensity profiles for the NOT operation using silicon microrings waveguide at 1.55 μm, demonstrating the following input states: (**a**) “0” and (**b**) “1”.

**Figure 12:**
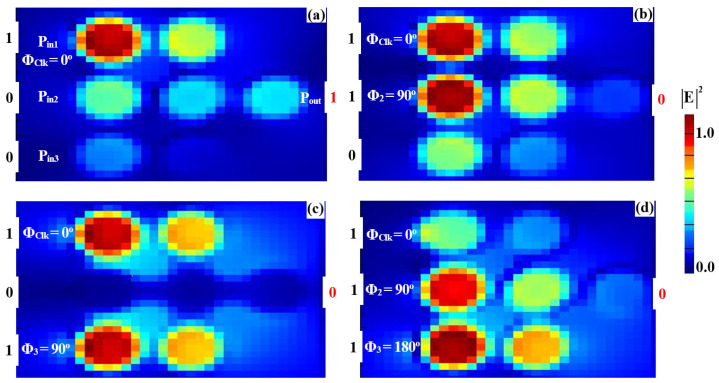
Field intensity profiles for the NOR operation using silicon microrings waveguide at 1.55 μm, demonstrating the following input states: (**a**) “00”, (**b**) “10”, (**c**) “01”, and (**d**) “11”.

**Figure 13:**
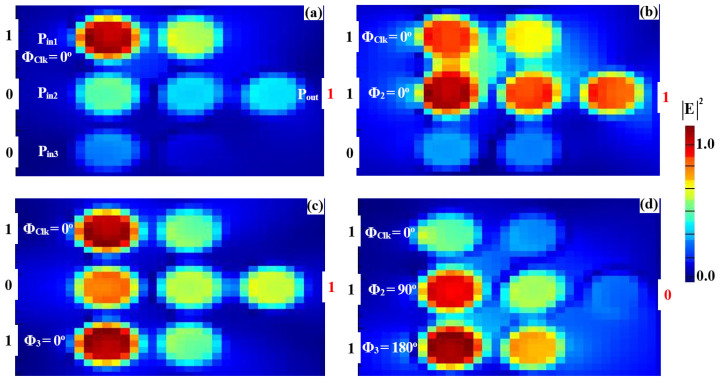
Field intensity profiles for the NAND operation using silicon microrings waveguide at 1.55 μm, demonstrating the following input states: (**a**) “00”, (**b**) “10”, (**c**) “01”, and (**d**) “11”.

**Figure 14:**
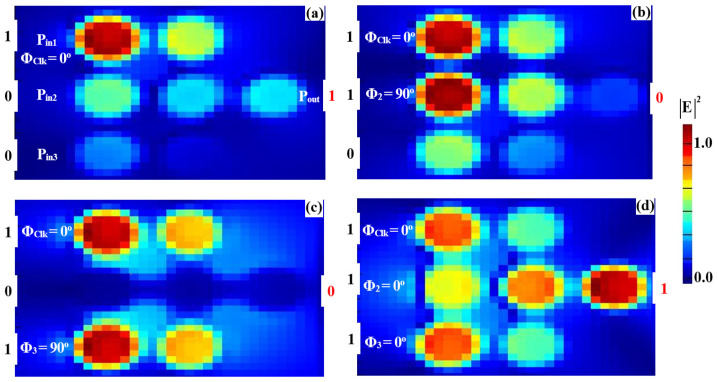
Field intensity profiles for the XNOR operation using silicon microrings waveguide at 1.55 μm, demonstrating the following input states: (**a**) “00”, (**b**) “10”, (**c**) “01”, and (**d**) “11”.

The simulated field distributions for the NOT, NOR, NAND, and XNOR logic gates, based on our silicon microring waveguides, are shown in Figure 11, Figure 12, Figure 13 and Figure 14, with corresponding performance metrics detailed in Table 3. These figures demonstrate how the clock signal (Clk) and the phase angles of input beams affect the output logic states. The Clk signal is injected into P_in1_, while the other input signals are applied to P_in2_ and P_in3_. In the case of the NOT, NOR, NAND, and XNOR gates, the proper synchronization of the input signals with the Clk signal is essential for ensuring accurate logic state determination. Phase misalignment can significantly impact the interference patterns, leading to incorrect logic outputs. Although phase control can be challenging, particularly in integrated photonic systems, our design ensures reliable phase synchronization by using a clock-driven mechanism that maintains stable interference conditions. This approach reduces phase-induced errors, guaranteeing precise logic operations in all cases. For the NOT gate (Figure 11), when only Clk (a continuous stream of logical 1s) is applied at Φ_Clk_ = 0°, the field intensity at P_out_ reaches 0.462, corresponding to a logical “1” (Figure 11a). However, when Clk and the input beam are injected at different angles (e.g., Φ_Clk_ = 0° and Φ_3_ = 90°), DI reduces the field intensity at P_out_ to 0.021, resulting in a logical “0” (Figure 11b). In the NOR gate (Figure 12), input beams with varying phase angles produce field intensities at P_out_ of 0.028 and 0.034, both representing a logical “0”. For the NAND gate (Figure 13), when the input beams are aligned (Φ_Clk_ = Φ_2_ = Φ_3_ = 0°), CI increases the field intensity at Pout to 0.672, representing a logical “1.” However, misaligned phase angles reduce the intensity to 0.034, indicating a logical “0”. The XNOR gate (Figure 14) produces a logical “1” when the input beams have identical angles, resulting in a CI field intensity of 0.886 at P_out_. When the input beams are set at different angles, the field intensity drops below the threshold (Tth), yielding a logical “0”. Table 3 summarizes these results, underscoring the capability of silicon microring waveguides to perform efficient and precise NOT, NOR, NAND, and XNOR logic operations. This confirms their suitability for integration into cutting-edge photonic circuits.

**Figure 15:**
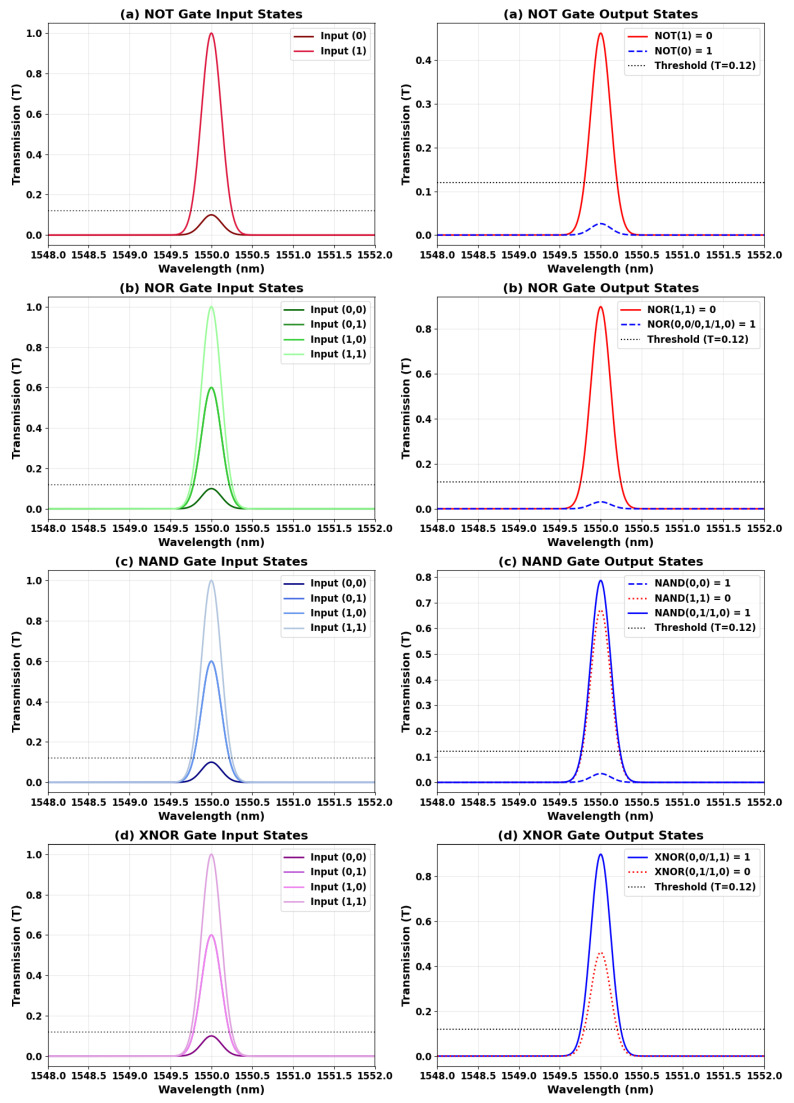
Transmission spectra of (**a**) NOT, (**b**) NOR, (**c**) NAND, and (**d**) XNOR gates at 1.55 µm, showing input states (**left**) and corresponding outputs (**right**). The dashed gray line indicates the logic threshold (Tth = 0.12).

Figure 15 illustrates the input–output spectral relationships of four inverted logic gates—NOT, NOR, NAND, and XNOR—implemented using a microring resonator platform operating at 1.55 µm. The left panels depict the intensity and phase combinations of the input states, while the right panels show the corresponding output spectra. The NOT gate exhibits inversion behavior, converting a logical “0” to a high transmission level (T = 0.462) and a logical “1” to a suppressed output (T = 0.026), achieving a contrast ratio of 12.5 dB. The NOR gate produces a strong output only for the “00” input state (T = 0.898), with all other states effectively suppressed, yielding a contrast of 14.6 dB. For the NAND gate, high transmission levels (T = 0.672–0.786) are maintained for all input combinations except “11”, which results in significant attenuation (T = 0.034), corresponding to a contrast of 12.9 dB. The XNOR gate responds strongly to matched inputs (“00” and “11”) with peak output transmission (T = 0.898), while mismatched states (“01” and “10”) are suppressed (T = 0.031), achieving a 14.6 dB contrast. All four gates demonstrate strong spectral alignment at 1.55 µm and consistently respect the defined threshold (Tth = 0.12), confirming the design’s ability to realize diverse logic functions through interference control. The high contrast ratios (>12 dB) further ensure cascadability and suitability for scalable photonic computing architectures.

## 4. Performance Comparison

Our study highlights significant advancements in CR values compared to prior works, as summarized in Table 4. The silicon microring waveguides in this study, with a compact size of 1.30 × 1.35 μm^2^, achieve simulated CRs between 12.02 and 15.85 dB across seven logic operations, including XOR, AND, OR, NOT, NOR, NAND, and XNOR. Unlike other designs that often rely on extinction ratio (ER) metrics, such as silicon micro-ring resonators (ER ≈ 10 dB, experimental) [16], our focus on CR provides a more comprehensive characterization of logic-level dynamics. These results outperform prior designs such as photonic crystal (PhC) waveguides (CR = 5.42–9.59 dB, simulation) [22], plasmonic logic gates (CR = 4.14–14.46 dB, simulation) [29], and inverse-designed silicon platforms (CR = 0.5–5.79 dB, simulation) [30]. They are competitive with metal slot waveguides (CR = 6–16 dB, experimental) [27]. The superior CR values in our design can be attributed to several factors that enhance light–matter interaction and optimize the resonance characteristics. First, the compact geometry (1.30 × 1.35 μm^2^) of the silicon microring waveguides plays a significant role in improving the confinement of light within the waveguide. This tight confinement, combined with optimal coupling between the waveguide and the microring, ensures that the energy transfer is maximized at resonance. As a result, constructive interference strengthens the desired signal, while destructive interference effectively suppresses unwanted signals, leading to higher CR values. Second, the precise interference control achieved through fine-tuning of the microring resonances is a crucial factor. By carefully controlling the resonance wavelengths, we ensure that the logic gates produce sharp transitions between logic states, which enhances the CR. This ability to precisely tune the resonance wavelengths is a key advantage over other designs, which may experience broader, less-defined transitions. Additionally, the high refractive index contrast of the silicon-on-silica material system allows for stronger light confinement within the waveguide, minimizing propagation losses and crosstalk between neighboring waveguides. This reduction in losses and crosstalk ensures that the logic gate operations are more reliable and result in higher CR values. The combination of these factors—compact structure, enhanced light confinement, and precise resonance control—enables our design to achieve superior performance in terms of CR. Our approach leverages superior light confinement, compact structure, and precise interference control to ensure high reliability and reduced latency for logic operations. By achieving high CR values and compactness, the proposed silicon microring waveguides contribute to scalable, high-performance optical networks and integrated photonic systems, paving the way for advancements in optical communication and computing technologies.

## 5. Data Rate Performance

The data rate of the system is calculated using the Nyquist formula, expressed as 2B log_2_[M], where M is the number of signal levels and B represents the optical bandwidth [39]. The bandwidth is determined by the equation B = (c/λ^2^) Δλ, where c is the speed of light in a vacuum, λ = 1.55 μm is the optical carrier wavelength, and Δλ is the spectral width of the signal [9,10]. In this study, the optical bandwidth is calculated as 49.95 GHz, with M = 4 (indicating the four possible signal states: 00, 10, 01, and 11). This results in a data rate of 199.8 Gb/s. This data rate of 199.8 Gb/s is a significant achievement, reflecting the system’s ability to handle high-speed data transmission, which is critical for modern telecommunications and data networks. By demonstrating the capability to perform optical logic operations at this speed, the proposed waveguide design shows promise for deployment in high-performance optical communication and computing systems. The ability to process data at such a high rate enhances throughput and reduces latency, leading to a more efficient and responsive system. Furthermore, the successful implementation of optical logic operations at this speed proves the scalability and robustness of the waveguide design, making it suitable for applications in future data centers, optical interconnects, and next-generation computing systems. This represents a significant advancement in optical communication technology, offering faster, more efficient, and reliable solutions for data processing.

## 6. Fabrication Feasibility and Performance Characteristics

The fabrication of functional ring resonators with outer radii (b) of 0.2 μm and inner radii (a) of 0.13 μm has been successfully demonstrated using deep ultraviolet (DUV, 193 nm ArF excimer laser) and extreme ultraviolet (EUV, 13.5 nm) lithography techniques [40,41]. Recent advancements in electron-beam lithography (EBL) and silicon-on-insulator (SOI) technology have enabled the realization of sub-100 nm silicon photonic structures, achieving critical dimension control with ±5 nm uniformity [42,43]. While there has been significant progress, current fabrication technologies do not yet support the fabrication of silicon photonic waveguides with the precise aspect ratio of 70 nm width and 300 nm height as used in our simulations. We have made a clear note in the manuscript stating that the experimental realization of such devices remains a challenge to be addressed in the future, but is conceptually feasible with further advances in high-precision EBL and related technologies. Recent studies have confirmed the feasibility of fabricating silicon nanostructures as narrow as 70 nm in width and 300 nm in height using advanced fabrication techniques such as high-precision EBL and self-aligned spacer lithography [44,45]. These ultracompact resonators exhibit distinct performance characteristics, with typical quality factors (Qs) in the range of 80–120, which are limited by radiative bending losses that scale exponentially with decreasing radius [3]. Meanwhile, free spectral ranges (FSRs) exceed 450 nm in the C-band (1530–1565 nm), as verified by spectral measurements of fabricated devices [46]. However, extinction ratios (ER) are compromised, reducing to 3–5 dB compared to >20 dB in conventional microrings, primarily due to insufficient evanescent field overlap in coupling regions [47]. To address these limitations, three key strategies have been developed: (1) hybrid plasmonic–dielectric configurations, incorporating silver (Ag) or aluminum (Al) cladding layers, significantly enhance mode confinement, achieving Q > 150 while maintaining sub-λ³ modal volumes [48]; (2) elliptical racetrack geometries that reduce bending losses by 40% at critical 90° turns compared to traditional circular designs [49]; and (3) inverse-designed couplers, optimized through computational techniques, which improve evanescent coupling efficiency by a factor of 2.8× [50]. Recent implementations of these approaches in silicon-on-insulator (SOI) platforms with 220 nm device layers have demonstrated successful applications in biosensing (bulk sensitivity = 180 nm/RIU) and dense wavelength division multiplexing (DWDM) (channel spacing = 400 GHz) [51,52]. These developments confirm the potential for ultracompact photonic devices and provide effective strategies for improving their performance in future implementations.

## 7. Conclusions

In conclusion, the proposed silicon microring resonator design demonstrates significant advancements in all-optical logic gate performance, achieving high contrast ratios (CR) for XOR (14.04 dB), AND (15.14 dB), OR (15.85 dB), NOT (13.42 dB), NOR (12.02 dB), NAND (12.75 dB), and XNOR (14.10 dB) gates. These values surpass those achieved by previous designs, ensuring enhanced signal integrity and operational efficiency. The compact waveguide size of 1.30 × 1.35 μm^2^, coupled with the high-quality factor of the microring resonators, results in a data rate of 199.8 Gb/s, demonstrating the capability of the proposed design to meet the growing demands for high-speed data transmission in optical communication and computing systems. This work underscores the potential of microring resonators and silicon photonics in realizing scalable, energy-efficient, and high-performance optical networks and integrated circuits, paving the way for future advancements in optical logic and communication technologies.

## Figures and Tables

**Table 1:** Mesh convergence study results for FDTD simulations, showing the impact of varying x- and y-direction mesh sizes (with fixed z-spacing of 10 nm) on transmission, propagation loss, and relative computational cost.

Mesh Size (nm)	Transmission	Loss (%)	Relative Cost *
5	0.8934	10.66	~50×
10	0.8936	10.64	~25×
20	0.8951	10.49	~10×
30	0.8911	10.89	~5×
40	0.8940	10.60	~2×
**50**	**0.8980**	**10.20**	~**1×**

* Relative cost estimate (50 nm = 1× baseline).

**Table 2:** Overview of XOR, AND, and OR logic gate performance using silicon microrings waveguide at a wavelength of 1.55 μm, with a threshold transmission (Tth) of 0.12.

Logic Operation	Input Signals			Output Logic	P_out_ (mW)	T	CR (dB)
	P_in1_	P_in2_ (Clk)	P_in3_	P_out_			
**XOR**	0	1	0	0	0.0074	0.031	**14.04**
	1	1	0	1	0.1886	0.786	
	0	1	1	1	0.1886	0.786	
	1	1	1	0	0.0074	0.031	
**AND**	0	1	0	0	0.0074	0.031	**15.14**
	1	1	0	0	0.0069	0.029	
	0	1	1	0	0.0062	0.026	
	1	1	1	1	0.2155	0.898	
**OR**	0	1	0	0	0.0074	0.031	**15.85**
	1	1	0	1	0.1886	0.786	
	0	1	1	1	0.1886	0.786	
	1	1	1	1	0.2155	0.898	

**Table 3:** Overview of NOT, NOR, NAND, and XNOR logic gate performance using the silicon microrings waveguide at a wavelength of 1.55 μm, with a threshold transmission (Tth) of 0.12.

Logic Operation	Input Signals			Output Logic	P_out_ (mW)	T	CR (dB)
	P_in1_ (CLK)	P_in2_	P_in3_	P_out_			
**NOT**	1	-	0	1	0.1109	0.462	**13.42**
	1	-	1	0	0.0050	0.021	
**NOR**	1	0	0	1	0.1109	0.462	**1** **2.02**
	1	1	0	0	0.0077	0.032	
	1	0	1	0	0.0050	0.021	
	1	1	1	0	0.0082	0.034	
**NAND**	1	0	0	1	0.1109	0.462	**12.75**
	1	1	0	1	0.1886	0.786	
	1	0	1	1	0.1613	0.672	
	1	1	1	0	0.0082	0.034	
**XNOR**	1	0	0	0	0.1109	0.462	**1** **4.10**
	1	1	0	0	0.0077	0.032	
	1	0	1	0	0.0050	0.021	
	1	1	1	1	0.2155	0.898	

**Table 4:** Performance comparison of optical logic operations in the proposed and existing waveguide architectures.

Logic Operations	Waveguide	Materials	Size (μm^2^)	λ (nm)	Metric (dB)	Experimental/ Simulation	Ref.
AND, NOR, XNOR	Si photonics platform	Si	~3 μm-long	1550	CR > 10	Exp.	[11]
AND, NAND	Silicon micro-ring resonators	Si/SiO_2_	0.45 × 0.25	1550.7	ER~10	Exp.	[16]
AND, XOR, OR, NOT, NAND, NOR XNOR	PhC waveguides	Si/Air	9 × 5	1550	CR = 5.42–9.59	Sim.	[22]
AND, XOR, XNOR	T-shaped PhC waveguides	Si/Air	-	1550	CR = 8.29–33.05	Sim.	[23,24,25]
AND, OR	2D PhC design	Si /Air	19.8 × 12.6	1520	CR = 9.74 and 17.95	Sim.	[26]
NOT, XOR, AND, OR, NOR, NAND, XNOR	Metal slot waveguide	Silver/SiO_2_	1.5 × 2.36	632.8	CR = 6–16	Exp.	[27]
NOT, XOR, AND, OR, NOR, NAND, XNOR	Metal–insulator–metal structures	Air/Silver	5.33 × 0.42	632.8	CR = 15	Sim.	[28]
AND, NAND, OR, XOR, NOR, XNAOR, NOT	Plasmonic logic gates design	Silver/SiO_2_	0.25 × 0.25	850	CR = 4.14–14.46	Sim.	[29]
AND, OR, NOT, NAND	Inverse design on silicon platforms	Si/SiO_2_	1.0 × 1.5	1300	CR = 0.5–5.79	Sim.	[30]
**XOR, AND, OR, NOT, NOR, XNOR, NAND**	**Silicon microrings waveguide**	**Si/SiO_2_**	**1.30 × 1.35**	**1550**	**CR = 12.02–15.85**	**Sim.**	**This work**

## Data Availability

Data are contained within the article.
